# Natural Nitrogenous Sesquiterpenoids and Their Bioactivity: A Review

**DOI:** 10.3390/molecules25112485

**Published:** 2020-05-27

**Authors:** De-Li Chen, Bo-Wen Wang, Zhao-Cui Sun, Jun-Shan Yang, Xu-Dong Xu, Guo-Xu Ma

**Affiliations:** 1 Institute of Medicinal Plant Development, Chinese Academy of Medical Sciences & Peking Union Medical College, No. 151, Malianwa North Road, Haidian District, Beijing 100193, China; chendeli9999@163.com (D.-L.C.); flydancingsun@163.com (Z.-C.S.); jsyang@implad.ac.cn (J.-S.Y.); 2 Hainan Branch of Institute of Medicinal Plant Development, Chinese Academy of Medicinal Sciences & Peking Union Medical College (Hainan Provincial Key Laboratory of Resources Conservation and Development of Southern Medicine), Haikou 570311, China; 3 School of Chemical Engineering and Technology, Hainan University, Haikou 570228, China; 13699719479@163.com

**Keywords:** nitrogenous sesquiterpenoids, celastraceae, marine sponge, fungi, bioactivities

## Abstract

Nitrogenous sesquiterpenoids fromnatural sourcesare rare, so unsurprisingly neither the potentially valuable bioactivity nor thebroad structural diversity of nitrogenous sesquiterpenoids has been reviewed before. This report covers the progressduring the decade from 2010 to February 2020 on the isolation, identification, and bioactivity of 391 nitrogen-containing natural sesquiterpenes from terrestrial plant, marine organisms, and microorganisms. This complete and in-depth reviewshouldbe helpful for discovering and developing new drugs of medicinal valuerelated to natural nitrogenous sesquiterpenoids.

## 1. Introduction

The natural products commonly termed ‘secondary metabolites’in contrast to ‘primary metabolites’, are produced byorganisms in order to provide an evolutionary benefit [1]. Natural products as a major chemical resource, have played a significantrole over the last 200 years in treating and preventing diseases, and continue to serve as important agents in modern drug discovery due to their characteristic chemical spatial orientation, which enables them tointeract with their natural and other biological targets [1,2,3,4]. Recently, half of new drugs reported were naturally occurring or constructed on the basis of some natural chemical framework [4,5,6].

Sesquiterpenoids are the largest class of natural terpenoids, with a structural diversity that includes thousands of compounds and more than 100 skeletaltypes [7]. Many of them show ‘drug-like’ chemical properties, including alkylating centerreactivity, lipophilicity, and favorable molecular geometry and electronic features, and have attractedconsiderable interest due to their pronounced biological activities [8,9]. Meanwhile, sesquiterpenoids that contain nitrogen bonds constitute a fascinating group with enormousstructural diversity [10]. Interestingly, it is notable that nitrogenous sesquiterpenoids are rare innatural sources, and there are only a few hundred such compounds that contain the element Nknown to be produced bycertain species. Functionally and biologically important to humans, have caught the attention of a number ofscientists, and extensive phytochemical and biologicalinvestigations of nitrogenous sesquiterpenoids from natural sources have been carried out by researchers at the recent ten years [10,11,12].

While the scientific community is generally aware of the rarity of the N bond in natural sequiterpenoids, and there are many reviews providing extensive coverage on sesquiterpenoids [11,12], including the naturally occurring disesquiterpenoids [1,13,14], natural products containing a nitrogen-nitrogen bond [15] or nitrogen-sulfur bond [16], neither the potentially valuable bioactivity nor the broad structural diversity of nitrogenous sesquiterpenoids has been systematically reviewed during the past ten years.

In this review, nitrogenous sesquiterpenoids from biological sources, including plants, microorganisms, and marine resources, will be considered. In order to be as comprehensive and clear as possible, the natural nitrogenous sesquiterpenoids have been segregated by structural class and compounds covered in the past decade included where appropriate. This report provides a systematic review of the isolation, structural characterization and biological activities of these compounds since 2010, if known.

## 2. Species Containing Nitrogenous Sesquite Rpenoids and Their Bioactivities

### 2.1. Dihydroagarofuran Sesquiterpenoids

**Figure 1:**
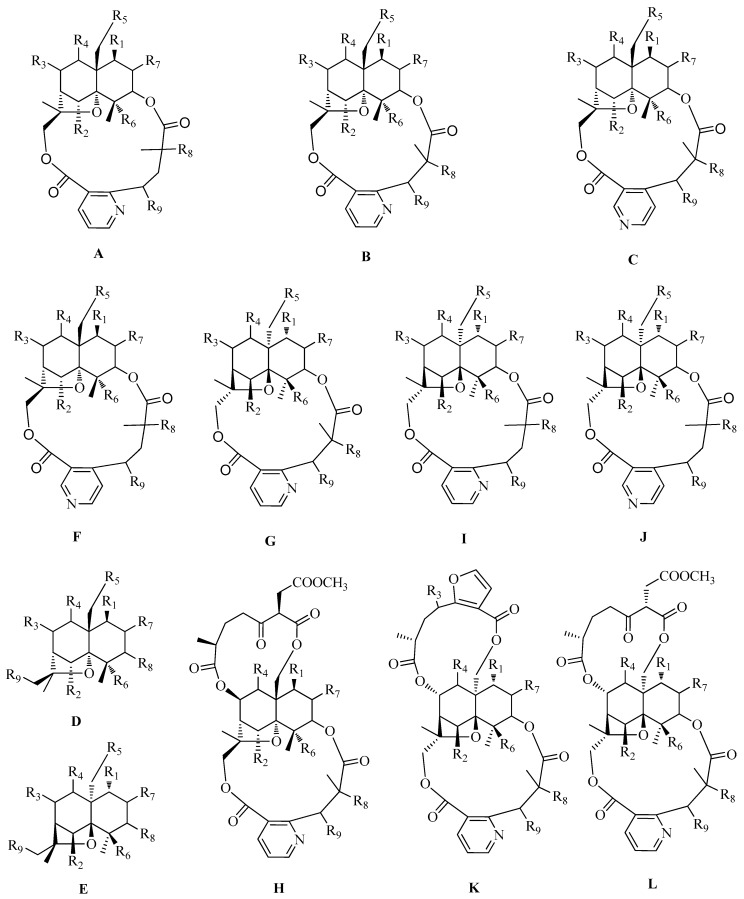
Twelve types (**A**–**L**) of dihydroagarofuran sesquiterpenoid skeletons.

Nitrogen-containing dihydroagarofuran sesquiterpenoids feature several ester groups on a highly oxygenated tricyclic scaffold, and their polyesterified macrolide sesquiterpenoid pyridine alkaloids possess a characteristic macrocyclic dilactone skeleton consisting of a dicarboxy licacid moiety, 2-(carboxyalkyl)nicotinic acid, and a polyoxygenated dihydro-β-agarofuran sesquiterpenoid (Figure 1 and Table 1). The hydroxyl groups of the latter are usually esterified by various organicacids including acetic, benzoic, furanoic, nicotinic, and cinnamicacids. The 2-(carboxyalkyl)nicotinic acid moiety originates from evoninic acid, wilfordic acid, hydroxywilfordic acid, ortheir congeners. The number, position, and configuration of these substituents create a largenovel chemical diversity and exhibit abroadrange of biological activities.

Dihydroagarofuran sesquiterpenoids were considered the most widespread and characteristic metabolites of the plants of the Celastraceae. Compounds **1**–**12** were isolated from the roots of *Maytenus mekongensis* [17]. Compounds **1**–**5** having wilfordic acid moieties, either with or without a 9′-OAc group, exhibited comparable antiplasmodial activities, with IC_50_ values of 3.1 × 10^−3^, 3.9 × 10^−3^, 3.5 × 10^−3^, 3.1 × 10^−3^ and 2.5 × 10^−3^ mM respectively, while compounds **10**–**12** with evoninic acid moieties showed no inhibitory activity. Compounds **12**–**29** were extracted from the dried roots of *Tripterygium wilfordii* [18]. Compound **22** displayed 22.3% inhibitory activity against HSV2 in vitro at 0.5 mg/mL, and acyclovir 66.3% inhibitory activity at 0.5 mg/mL. Compound **28** showed 31.7% inhibitory activity at 0.25 mg/mL, while acyclovir displayed 60.6% inhibitory activity at 0.25 mg/mL. Compounds **30** and **31** were obtained from the fruits of *Celastrus orbiculatus* Thunb [19]. Hypoglaunines E (**32**) and F (**33**) have been purified from the root barks of *Tripterygium hypoglaucum* and showed no cytotoxic activities against five cancer celllines [20]. Triptersinines A–H, L (compounds **34**–**42**), peritassine A (**26**), wilfordinine A (**43**), hypoglaunine A (**44**), hypoglaunine E (**32**), wilfordinine E (**45**), euonine (**46**), wilfortrine (**21**), euonymine (**12**) were extracted from the leaves of *Tripterygium wilfordii*, and compounds **26**, **34**, **43**, and **46** showed moderate inhibitory effects onnitric oxide production in LPS-induced macrophages at 5 μM [21]. Compounds **47**–**49** were identified from thestems of *Euonymus alatus* [22]. Triptersinines M–T (compounds **50**–**57**) and wilforgine (**18**) have been extracted from the the leaves of *Tripterygium wilfordii*, and compounds **50**, **51**, **54**, **57**, and **18** showed moderate inhibitory abilities on NO production and no influence on cell viability by the MTT method, the other compounds exhibited weak effects [23]. Compounds **7**, **25**, **58**–**91** were obtained from the dried roots of *Tripterygium wilfordii* [24]. Tripterygiumine Q (**81**) exhibited immunosuppressive activity with an IC_50_ value of 8.67 μM, and no cytotoxicity was observed even at a dose of 100 μM. Triptonine B (**82**) not only exhibited immunosuppressive activity with an IC_50_ value of 4.95 μM, but also showed cytotoxicity with an IC_50_ value of 26.41 μM. Compounds **92**–**95** were isolated from the leaves of *Maytenus spinosa* [25], and the isolates displayed no anti-HIV activity. Tripterygiumines S-W (**96**–**100**), wilfornine A (**101**), wilfornine D (**102**), tripfordine A (**103**), 2-debenzoyl-2-nicotinoylwilforine (**104**) along with **12**–**13**, **18**–**20**, **25**, **75**, and **87** were purified from the roots of the *Tripterygium wilfordii*, and found that **13** and **96** possessed potent nitric oxide inhibitory activity with IC_50_ values ranging from 2.99 to 28.80 μM, without any effect on the cell viability of RAW 264.7 cells [26]. Accordingly, compounds **13** and **96**, especially **13**, were identified as promising candidates for further scientific investigation of their potential use as anti-inflammatory agents. Compound **105** was obtained from the whole plants of *Parnassia wightiana*, and showed some cytotoxic activities against NB4, MKN-45 and MCF-7 cells at 20 μM [27]. Triptregelines A-J (**106**–**115**), regelidine (**28**), 1α,6β,15-triacetoxy-8α-benzoyloxy-4β-hydroxyl-9α-(3-nicotinoyloxy)-dihydro-β-agarofuran (**116**), dimacroregeline A-B (**117**–**118**) and triptonine A (**119**) have been isolated from the stems of *Tripterygium regelii*, and **107**, **108**, **113** and **116** exhibited weak cytotoxic effects on taxol-resistant A549T with IC_50_ values ranged from 29.4 to 54.4 μM [28], **118** showed inhibitory effects on the proliferation of human rheumatoid arthritis synovial fibroblast cell (MH7A) at a concentration of 20 µM [29]. Compounds **120**–**123** were extracted from the stems of *Maytenusoblongata* [30]. 1-*O*-Benzoyl-1-deacetyl-4-deoxyalatamine (**121**) and 1,2-*O*-dibenzoyl-1,2-deacetyl-4-deoxyalatamine (**122**) exhibited strong larvicidal activity on the *A. aegypti* Paea strain with LD_50_ values of 9.4 (95% CI: 6.5–10.0) and 2.7 μM (95% CI: 1.9–2.9), respectively. Triptersinine U (**124**), hypoglaunine B (**125**) together with **26**, **32**, **33**, **43**, **44**, and **46** were isolated from the roots of *Tripterygium wilfordii*, but all dihydroagarofuran derivatives didn’t show cytotoxicity against six human tumor celllines (HepG2, Hep3B, Bcap37, U251, MCF-7 and A549) [31]. Neuroprotective triptersinine Z4–Z14 (**126**–**130**, **132**–**137**) and euojaponine C (**131**) have been obtained from the leaves of *Tripterygium wilfordii* [32,33], and **126**, **127**, **129**–**131** increased cell viability of the okadaic acid-treated PC12 cells from 60.4 ± 23.0% to 72.4 ± 14.1, 71.5 ± 11.5, 75.7 ± 15.6,81.2 ± 13.1, and 86.2 ± 25.5% at 10 μM, respectively [32]. At 10 μmol/L, compounds **132** and **133** showed moderate inhibitory effects on NO production in LPS-induced macrophages with inhibitory rate at 31.2 ± 3.6 and 40.9 ± 4.3 [33]. Two new sesquiterpene pyridine alkaloids, Chinese bittersweet alkaloid A (**138**) and Chinese bittersweet alkaloid B (**139**) were isolated from the rootbarks of *Celastrus angulatus* [34]. Monimins I (**140**) and II (**141**) have been extracted from the leaves of *Monimopetalum chinense* [35]. Tripteryford C (**142**) and tripteryford E (**143**) have been obtained from the leaves of *Tripterygium wilfordii*, and **142** exhibited the better protective activity against human neuroblastoma SH-SY5Y cell injury induced by H_2_O_2_ with 76.63% cell viability comparing with the positive control Trolox (69.84%) at 12.5 μM [36]. Celaspaculin G (**144**) was purified fromthe seeds of *Celastrus paniculatus*, and with non lifespan-extending effect on the nematode *Caenorhabditis elegans* [37].

### 2.2. Drimane and Friedo-Drimane Sesquiterpenoids

**Figure 2:**
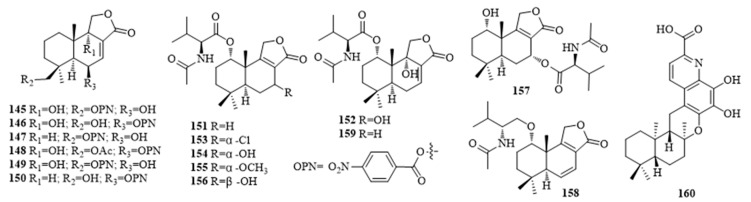
The structures of compounds **145**–**160**.

Nitrobenzoyl drimane sesquiterpenoids are rare in natural sources, *Aspergillus* fungi species being the only known sources.6β,9α-Dihydroxy-14-*p*-nitrobenzoylcinnamolide (**145**) and insulicolide A (**146**), insulicolide B (**147**), 14-*O*-acetylinsulicolide A (**148**), insulicolide C (**149**) and 9-deoxyinsulicolide A (**150**) (Figure 2) were isolated from extracts of the culture of marine-derived fungus *Aspergillus ochraceus* Jcma1F17 [38,39]. All of them displayed significant cytotoxicity against 10 human cancer celllines (H1975, U937, K562, BGC-823, Molt-4, MCF-7, A549, Hela, HL60, and Huh-7), with IC_50_ values ranging from 1.95 mM to 6.35 mM, and **145** also exhibited moderate inhibitory activity against two viruses, H3N2 and EV71, with IC_50_ values of 17.0 and 9.4 mM, respectively [38]. Compound **146** showed the strongest activities, with IC_50_ values of 1.5, 1.5, and 0.89 μM, against ACHN, OSRC-2, and 786-O cells, respectively [39]. **148** indicated potent inhibitory activities atlowμM levels, comparable to the positive control, sorafenib, adrug (Nexavar) approved for the treatment of primary kidneycancer (advanced renal cell carcinoma) [39]. Additionally, **145** and **148** exhibited stronger cytotoxicity to 786-*O* cells (IC_50_ 4.3 and 2.3 μM, respectively) than to OS-RC-2 (IC_50_ 8.2 and 5.3 μM, respectively) and ACHN (IC_50_ 11 and 4.1 μM, respectively) [39]. Purpuride (**151**), berkedrimane B (**152**), minioluteumides A–D (**153**, **154**, **156** and **157)**, purpuride B (**155**) (Figure 2) featuring with lactones conjugated a *N*-acetyl-L-valine, and such drimane sesquiterpenoid are rare in nature, which were extracted from the marine fungus, *Talaromyces minioluteus* (*Penicillium minioluteum*) [40]. Compounds **152**, **153** and **157** exhibited cytotoxic activity with IC_50_ values of 193.3, 50.6 and 57.0 µM against HepG2 cancer cell line, respectively [40]. A new sesquiterpene lactonepurpuride D (**158**), berkedrimane A (**159**), along with **151**, **152**, **155**, **157** (Figure 2) were prepared from a culture of marine-sourced fungus *Penicillum* ZZ1283 in the medium of potato dextrose broth was found to have antimicrobial activities with MIC values of 4–14 μg/mL against MRSA [41]. Saccharoquinoline (**160**) (Figure 2) composing of a drimane-type sesquiterpene unit in combination with anapparent 6,7,8-trihydroxyquinoline-2-carboxylic acidwithcytotoxicity against the HCT-116 cancer cell line by inducing G1 arrest, and was obtained from the fermentation broth of the marine-derived bacterium *Saccharomonospora* sp. CNQ-490 [42].

**Figure 3:**
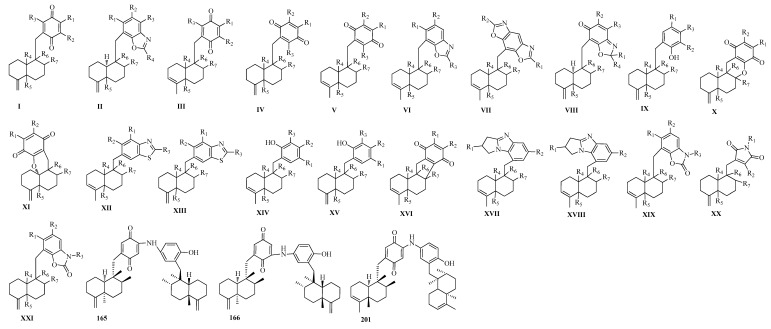
The friedo-drimane sesquiterpenoidskeletons (**I**–**XXI**) and three dimers.

Marine sponges are a rich source ofbioactive secondary metabolites, the majority of which are sesquiterpene quinones/hydroquinones, most of which possess either adrimane or a rearranged 4,9-friedodrimane terpenoid skeleton, which contains a C15 sesquiterpene moiety incorporating a C6 benzoquinone or hydroquinone group framework. Drimane sesquiterpene quinones represent a large group of biologically active marine natural products. Six nitrogenous drimane sesquiterpenoid aminoquinones (Figure 3 and Table 2), named 18-aminoarenarone (**161**), 19-aminoarenarone (**162**), 18-methylaminoarenarone (**163**), 19-methylaminoarenarone (**164**), along with two dimeric popolohuanone F (**165**), popolohuanone A (**166**) isolated from the Australian marine sponge *Dysidea* sp., and **165** and **166** showed DPPH radical scavenging activitywith IC_50_ values of 35.0 and 35.0 µM, respectively [43]. A new sesquiterpene benzoxazole, nakijinol B (**167**), its acetylated derivative, nakijinol B diacetate (**170**), and two newsesquiterpene quinones, smenospongines B (**168**) and C (**169**) (Figure 3 and Table 2), were extracted from the methanol extract of the marine sponge *Dactylospongia elegans*, and were found to have cytotoxic activities in the range of 1.8–46 µM against a panel of human tumor cell lines (SF-268, H460, MCF-7, and HT-29) and a normal mammalian cell line (CHO-K1) [44]. Investigation of the marine sponge *Dysideaavara*, three bioactive sesquiterpenoid Quinones afforded, (−)-3′-methylaminoavarone (**171**), (−)-4′-methylaminoavarone (**172**) and (−)-*N*-methylmelemeleone-A (**173**) (Figure 3 and Table 2) with their moderate protein kinase inhibition, cytotoxicity, inhibition of NFkB-activity and insecticidal activity [45]. Two sesquiterpene aminoquinines (Figure 3 and Table 2), smenospongine (**174**) and glycinylilimaquinone (**175**), were isolated from the Fijian marine sponge *Hippospongia* sp., and displayed lethality at LD_50_ = 188 and <500 ppmagainstbrineshrimp, respectively [46]. Bioactivity-guided isolation yielded fivenew sesquiterpene aminoquinones 5-*epi*-Nakijiquinone S-N (**176**–**180**), two new sesquiterpene benzoxazoles 5-*epi*-Nakijinol C–D (**181** and **182**) (Figure 3 and Table 2) isolated from the sponge *Dactylospongia metachromia* [47]. Compounds **176**–**180** showed potent cytotoxicity againstthe mouse lymphoma cell line L5178Y with IC_50_ values ranging from 1.1 to 3.7 μM [47]. When tested in vitro for their inhibitory potential against 16 different protein kinases, compounds **180** and **181** exhibited the strongest inhibitory activity against ALK, FAK, IGF1-R, SRC, VEGF-R2, Aurora-B, MET wt, and NEK6 kinases (IC_50_ 0.97–8.62 μM) [47]. Dysidaminones A-M (**183**–**195**) (Figure 3 and Table 2), thirteen new sesquiterpene aminoquinones, along with six known ones (**196**–**201**), were isolated from the South China Sea sponge *Dysidea fragilis* [48]. Compounds **185**, **187**, **190**, and **192**, **196**, and **198** showed cytotoxicity against mouse B16F10 melanoma and human NCI-H929 myeloma, HepG2 hepatoma, and SK-OV-3 ovarian cancer cell lines [48]. Inaddition, these six cytotoxic compounds also exhibited NF-kB inhibitory activity with IC_50_ values of 0.05–0.27 mM [48]. Four nitrogenous 4,9-friedodrimane-type sesquiterpenoids (**202**–**205**) (Figure 3 and Table 2) were acquired using the oxidative potential of *Verongula rigida* on bioactive metabolites from two *Smenospongia* sponges, and the mixture of **204** and **205** suppressed β-cateninresponse transcription (CRT) via degrading β-catenin and exhibited cytotoxic activity on colon cancer cells [49]. Compounds **206**–**214**, together with **174** (Figure 3 and Table 2) have been obtained from the Marine Sponge *Spongiapertusa* Esper, and **174**, **213**, **214** exhibited activities against the human cancer cell lines U937, HeLa, and HepG2, with most potent cytotoxicities to U937 cells with IC_50_ values of1.5, 2.8, and 0.6μM, respectively [50]. Four sesquiterpene hydroquinones, dactylospongins A–D (**215**–**218**), as wellas five sesquiterpene quinones, melemeleones B–E (**219**–**222**) and dysidaminone N (**223**) (Figure 3 and Table 2) were isolated from the marine sponge *Dactylospongia* sp., anti-inflammatory evaluation showed that **215**–**218**, and **223** exhibtited potent inhibitory effects on the production of inflammatory cytokines (IL-6, IL-1β, IL-8, and PEG2) in LPS-induced THP-1 cells with IC_50_ values of 5.1–9.2 μM [51]. A new sesquiterpenoid aminoquinone nakijiquinone V (**224**), along with smenospongine (**174**) (Figure 3 and Table 2) were extracted from an Indonesian marine *Dactylospongia elegans* sponge [52]. Eleven new nitrogenous meroterpenoids, cinerols A–K (**225**–**235**) (Figure 3 and Table 2), were isolated from the marine sponge *Dysideacinerea*, **225** and **226** feature a rare 5*H*-pyrrolo[1,2a]-benzimidazole moiety, while cinerols **227**–**231** were examples of rare meroterpene benzoxazoles [53]. Six sesquiterpene quinones/hydroquinones (**236**–**240**, **210**) (Figure 3 and Table 2) were acquired from the marine sponge Dactylospongia elegans [54]. Compounds **238**–**240** showed activities against the human cancer cell lines DU145, SW1990, Huh7, and PANC-1 with IC_50_ values ranging from 2.33 to 37.85 μM [54]. Three cytotoxic sesquiterpenoid quinones (**241**–**243**) (Figure 3 and Table 2) were purified from South ChinaSea sponge *Dysidea* sp., and displayed various potent cytotoxic activities with IC_50_ values ranging from 0.93 to 4.61 μM [55]. Two unique nitrogenous sesquiterpene quinone meroterpenoids, dysidinoid B (**244**) and dysicigyhone A (**245**) (Figure 3 and Table 2) were characterized from the marine sponge *Dysideaseptosa*, and 244) exhibited signifcant anti-inflammatory effect by inhibiting TNF-α and IL-6 generation with IC_50_ values of 9.15 μM and17.62 μM, respectively [56]. Two nitrogenous merosesquiterpene, 5-epi-nakijiquinone L (**246**) and 5-*epi*-smenospongiarine (**247**) (Figure 3 and Table 2) were isolated from the sponge *Verongula* cf. *rigida* with weak 5α-reductase inhibitory activity [57].

**Figure 4:**
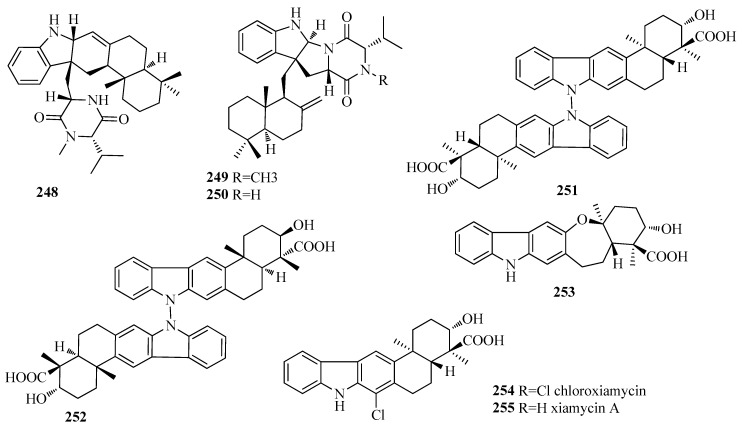
The structures of compounds **248**–**255**.

Drimane sesquiterpenoid-indole alkaloids rarely occur in Nature. Only eight compounds were isolated from actinomycete *Streptomyces* sp. Three hybrid isoprenoid drimane derivatives―indotertine A (**248**), drimentine F (**249**) and drimentine G (**250**) (Figure 4)—were afforded from a reed rhizosphere soil-derived actinomycete *Streptomyces* sp. CHQ-64 [58]. Compound **250** showed strong cytotoxicity against human cancer cells lines with IC_50′_s down to 1.01 μM, while **248** and **249** showed no significant activity [58]. Four new indolo-drimanesesquiterpenes, dixiamycins A (**251**) and B (**252**), oxiamycin (**253**), and chloroxiamycin (**254**), were isolated from a marine-derived actinomycete *Streptomyces* sp. and characterized, together with the known compound xiamycin A (**255**) (Figure 4) [59]. **251** and **252** are the first examples of atropisomerism of naturally occurring N−N-coupled atropo-diastereomers, with a dimeric indolo-sesquiterpene skeleton and a stereogenic N−N axis between sp^3^-hybridized nitrogen atoms [59]. The two dimeric compounds **251** and **252** showed better antibacterial activities than the monomers **253**–**255** with the IC_50_ values of 4–16 μg/mL against four indicator strains (*Escherichia coli* ATCC25922, *Staphylococcus aureus* ATCC 29213, *Bacillus subtilis* SCSIO BS01 and *Bacillus thuringiensis* SCSIO BT01) [59].

### 2.3. Eudesmane Sesquiterpenoids

**Figure 5:**
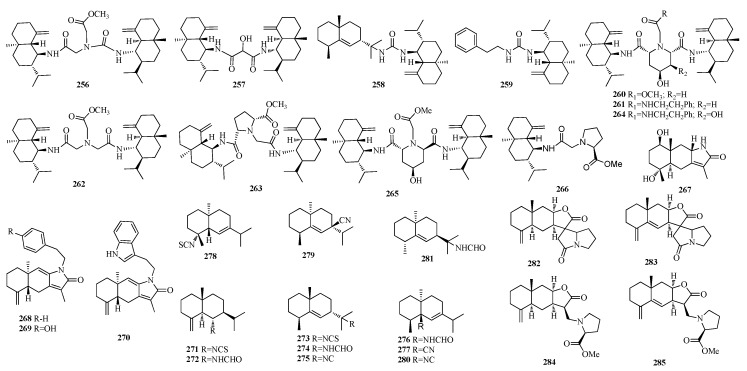
The structures of compounds **256**–**285**.

Eleven nitrogen-containing eudesmane sesquiterpenoids, halichonadins G–Q (**256**–**266**) (Figure 5), were isolated from a marine sponge *Halichondria* sp., and compounds **256** and **258** showed cytotoxicity against murine lymphoma L1210 cells (IC_50_ 5.9 and 6.9 μg/mL)and human epidermoid carcinoma KB cells (IC_50_ 6.7 and 3.4 μg/mL) in vitro, Halichonadin K showed cytotoxicity against human epidermoid carcinoma KB cells (IC_50_ 10.6 μg/mL) in vitro, and halichonadin O displayed antimicrobial activity against *Staphylococcus aureus* (MIC 8 µg/mL), *Micrococcus luteus* (MIC 8 µg/mL), and *Trichophyton mentagrophytes* (IC_50_ 16 µg/mL) [60,61,62]. One eudesmane-type sesquiterpene, phaeusmane I (**267**) (Figure 5), was isolatedfrom the rhizomes of *Curcuma phaeocaulis* [63]. Three new nitrogen-containing sesquiterpenoids, the cespilamides C–E (**268**–**270**, Figure 5) were purified from the Taiwanese soft coral *Cespitularia taeniata*, and **270** exhibited cytotoxicity against human breast adenocarcinoma (MCF-7), medulloblastoma (Daoy), and cervical epitheloid carcinoma (Hela) cancer cells with IC_50_ of 17.5, 22.3, and 24.7 μM, respectively [64]. Acanthine B (**271**), acanthine C (**272**), 11-isocyano-7β*H*-eudesm-5-ene (**273**), 11-isothiocyano-7β*H*-eudesm-5-ene (**274**), and 11-formamido-7β*H*-eudesm-5-ene (**275**) (Figure 5), were isolated from the Thai sponge *Halichondria* sp. [65]. Four new uncommon nitrogenous eudesmane-type sesquiterpenes, axiriabilines A–D (**276**–**279**), and one known related ent-stylotelline (**280**) (Figure 5), were isolated from the Hainan sponge *Axinyssa variabilis* with no cytotoxicity against several cancer cells [66]. Axiriabiline A (**276**) and 11-formamido-7β*H*-eudesm-5-ene (**281**) (Figure 5) were extracted from South China Sea Nudibranchs *Phyllidiella* sp. [67]. Spiroalanpyrroids A (**282**) and B (**283**), two sesquiterpene alkaloids with an unprecedented eudesmanolide-pyrrolizidine spiro [55] framework, were isolated together with two new sesquiterpene-amino acidadducts, helenalanprolines A (**284**) and B (**285**) (Figure 5), from the roots of *Inula helenium* [68]. Bioassays showed that **284** and **285** significantly inhibited nitric oxide production in lipopolysaccharide-induced RAW 264.7 macrophages with IC_50_ values of 15.8 and 13.5 μM, respectively [68].

### 2.4. Cadinane Sesquiterpenoids

**Figure 6:**
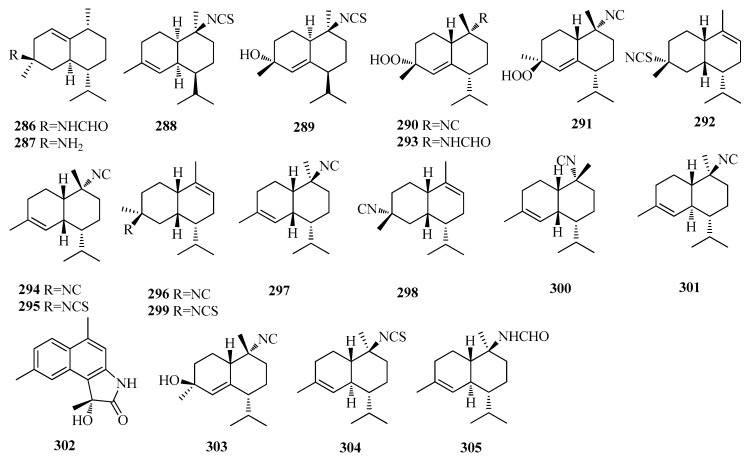
The structures of compounds **286**–**305**.

Two nitrogenous cadinane sesquiterpenes (3S*, 5R*, 6R*, 9R*)-3-formamido-1(10)-cadinene (**286**) and (−)-halichamine (**287**) (Figure 6) were isolated from the Thai marine sponge *Halichondria* sp. [69]. Compound **286** showed moderate cytotoxic activity against HeLa, MOLT-3, and HepG2 cell lines with IC_50_ valued of 32.1, 33.4, and 16.0 mM, respectively, while compound **287** also displayed moderate cytotoxic activity against HuCCA-1, MOLT-3, HepG2, and MDA-MB231 cell lines with IC_50_ valued of 20.3, 34.6, 19.9, and 22.6 mM, respectively [69]. (1*R*, 6*S*, 7*S*, 10*S*)-10-isothiocyanato-4-amorphene (**288**), axinisothiocyanate J (**289**) (Figure 6) were extracted from the marine sponge *Axinyssa* sp. [70]. Halichon C (**290**) and 4-epihalichon C (**291**), halichon D (**292**), halichonG (**293**), (−)-10-isocyano-4-cadinene (**294**), and (−)-10-isothiocyanato-4-cadinene (**295**) (Figure 6), were obtained from the Thai sponge *Halichondria* sp. [65]. Compounds **290**, **291**, and **294** exhibited moderate cytotoxicity (IC_50_ 20.9, 29.0, and 9.1 μM, respectively) against the MOLT-3 cell line and compound **292** also showed moderate cytotoxicity against HepG2 and MDA-MB-231 cell lines with IC_50_ values of 24.3 and 19.3 μM, respectively [65]. New stereoisomers of (+)-(1S*, 4S*, 6S*, 7R*)-4-Isocyano-9-amorphene (**296**) and of (−)-(1S*, 6R*, 7R*, 10S*)-10-isocyano-4-amorphene (**297**), 4α-isocyano-9-amorphene (**298**), (1S*, 4S*, 6S*, 7R*)-4-thiocyanate-9-cadinene (**299**), (−)-10-isocyano-4-amorphene (**300**), (−)-10-isothiocyanato-4-cadinene (**301**) (Figure 6), were identified from *Phyllidiella pustulosa* and from *Phyllidia ocellata* [71]. A novel sesquiterpenoidal lactam, commipholactam A (**302**) (Figure 6) was isolated from *Resina commiphora* [72]. Biological assessment against human cancer cells showed that the IC_50_ values of **302** against HepG2 and A549 cells were 21.73 μM and 128.50 μM, respectively [72]. Axidaoisocyanate A (**303**), 10-isothiocyanato-4-cadinene (**304**), 10-formamido-4-cadinene (**305**), along with **289**, **293** (Figure 6), were identified from two South China Sea Nudibranchs *Phyllidiella pustulosa*, *Phyllidia coelestis* [67].

### 2.5. Bisabolane Sesquiterpenoids

**Figure 7:**
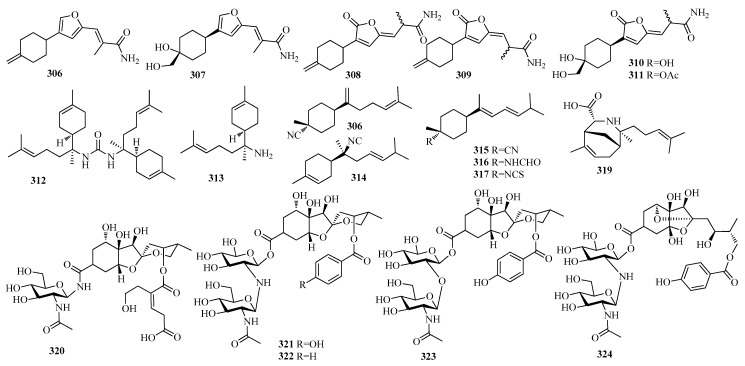
The structures of compounds **306**–**324**.

Brasilamides E–J (**306**-**311**), bisabolane sesquiterpenoids with 3-cyclohexylfuran (**306** and **307**) and 3-cyclohexylfuranone (**308**–**311**) skeletons (Figure 7), were isolated from scaled-up fermentation cultures of the plant endophytic fungus *Paraconiothynium brasiliense* Verkley [73]. Compound **307** selectively inhibited the proliferation of the breast (MCF-7) and gastric (MGC) cancer cell lines, with IC_50_ values of 8.4 and 14.7 μM, respectively [73]. N,N’-bis[(6*R*,7*S*)-7-amino-7,8-dihydro-a-bisabolen-7-yl]urea (**304**), and (6*R*,7*S*)-7-amino-7,8-dihydro-α-bisabolene (**313**) (Figure 7), were purified from the marine sponge *Axinyssa* sp. collected atIriomote Island [70]. Compound **312** was the most potent inhibitor of PTP1B activity (IC_50_ = 1.9 μM) without cytotoxicity at 50 μM in two human cancer cell lines, hepatoma Huh-7 and bladder carcinoma EJ-1 cells [70]. Compound **312** also moderately enhanced the insulin-stimulated phosphorylation levels of Aktin Huh-7 cells [70]. D^7,14^-3-isocyanotheonellin (**314**) and 3-isocyanotheonellin (**315**), theonellin formamide (**316**), theonellin isothiocyanate (**317**), and 7-isocyano-7,8-dihydro-α-bisabolene (**318**) (Figure 7) were extracted from the two South China Sea nudibranchs *Phyllidiella pustulosa* and *Phyllidia coelestis* [67]. Compounds **315**, **317**, and **318** exhibited strong cytotoxicity against human cancercell line SNU-398 with IC_50_ values of 0.50, 2.15, and 0.50 μM, respectively [67]. In addition, compound **315** also displayed broad cytotoxicity against the other three cancer cell lines, including A549, HT-29, and Capan-1, with IC_50_ values of 8.60, 3.35, and 1.98 µM, respectively [67]. A rearranged bisabolene-type sesquiterpene, halichonic acid (**319**), was isolated from a marine sponge *Halichondria* sp., together with**313 [74]** (Figure 7). Compound **313** was cytotoxic against HeLa cells with an IC_50_ value of 50 μM, whereas **314** did not show cytotoxicity even at 50 μM [74]. Five novel highly oxygenated norbisabolane sesquiterpene, namely phyllanthacidoid U (**320**), phyllanthacidoidA (**321**),phyllanthacidoid B (**322**), phyllanthacidoid L (**323**), and phyllanthacidoid S (**324**) (Figure 7) were isolated from the roots and stems of *Phyllanthus acidus*, and compounds **321**–**323** displayed potential anti hepatitis B virus (anti-HBV) activities [75].

### 2.6. Germacrane, Elemaneand Iresane Sesquiterpenoids

**Figure 8:**
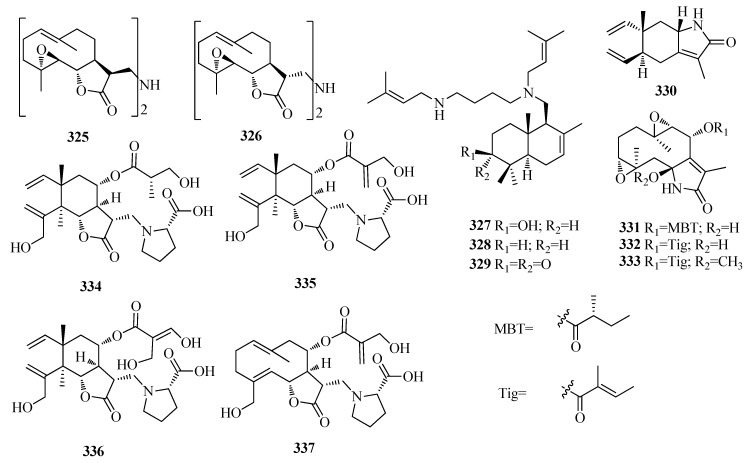
The structures of compounds **325**–**337**.

Two germacrane-type sesquiterpenoid dimers―isobisparthenolidine (**325**) and bisparthenolidine (**326**) (Figure 8) were isolated from the chloroform-soluble fraction of the methanolic extract of the bark of *Magnolia kobus* (Magnoliaceae) [76]. Compound **325** displayed broad cytotoxicity against four cancer cell lines, including A549, SK-OV-3, SK-MEL-2, and HCT-15, with IC_50_ values of 2.0, 1.9, 3.9 and 3.2 µM, respectively [76]. Noveliresane sesquiterpene alkaloids, halichonines A (**327**), B (**328**), and C (**329**) (Figure 8), were identified from the marine sponge *Halichondria okadai* Kadota, and **328** wasthen subjected to the trypan blue dye exclusion using HL60 human leukemia cells, and showed cytotoxicity (IC_50_ value: 0.60 µg/mL) [77]. One γ-elemene-type sesquiterpenes, 8β(*H*)-elema-1,3,7(11)-trien-8,12-lactam (**330**) (Figure 8) was obtained from the rhizomes of Curcuma phaeocaulis [63]. Three new germacrane sesquiterpenoid-typealkaloids with an unusual Δ^8^-7,12-lactam moiety, glechomanamides A–C (**331**–**333**) (Figure 8) were isolated from *Salvia scapiformis* [78]. In a tube formation assay, **332** showed the most potent antiangiogenic activity in primary screening, and its IC_50_ value was determined to be 40.4 μM [78]. In addition to VEGFR2, **332** decreased BMP4 expression, which regulates tube formation, and glycolysisrelated proteins, including GLUT1 and HK2, which suggests that the novel compound **332** is worthy of additional investigation for angiogenesis-associated pathological conditions [78]. Onopornoids A–D (**334**–**337**) (Figure 8), three elemanes and one germacrane, were extracted from the whole aerial parts of *Onopordum alexandrinum*, which possess unique structures combining a sesquiterpenoid framework with an amino acid, L-proline [79].

### 2.7. Farnesane, Spiroaxane, Aromadendrane and Pupukeanane Sesquiterpenoids

**Figure 9:**
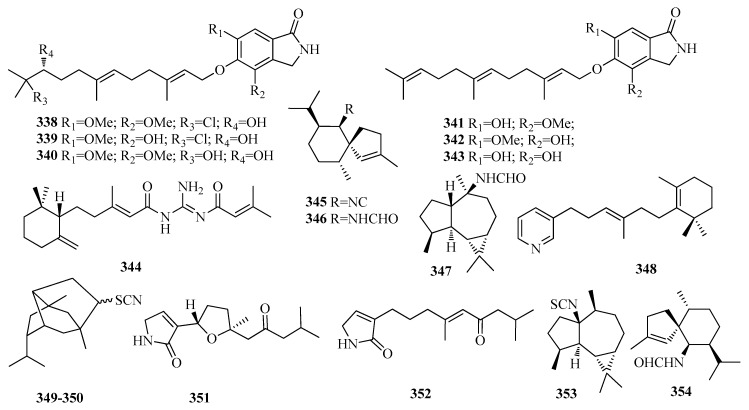
The structures of compounds **338**–**354**.

Chemical investigation of the endophytic fungus *Emericella* sp. (HK-ZJ) isolated from the mangrove plant *Aegiceras corniculatum* led to the isolation of six farnesane sesquiterpenoids named emeriphenolicins A–F (**338**–**343**) (Figure 9) with moderate anti-influenza A viral (H1N1) activities [80]. An unusual farnesane natural product (dotofide, **344**) (Figure 9), in which the terpenoid skeleton is interrupted by a guanidine moiety was obtained from the marine slug *Doto pinnatifida* [81]. Two spiroaxane sesquiterpenes, (−)-axisonitrile-3 (**345**), (+)-axamide-3 (**346**), and one aromadendrane sesquiterpene axamide-2 (**347**) (Figure 9) were isolated from the Thai marine sponge *Halichondria* sp., and only **345** showed strong activity to the HepG2 cell line withan IC_50_ value of 1.3 µM [69]. Fasciospyrinadine (**348**) (Figure 9), a novel farnesane sesquiterpene pyridine alkaloid was extracted froma Guangxi sponge *Fasciospongia* sp. [82].

Apupukeanane-type sesquiterpenoid isomers, 9-thiocyanatopupukeanane isomers (**349**–**350**) (Figure 9) were isolated from the the Thai sponge *Halichondria* sp. [65]. A bioassay-guided phytochemical study was conducted on the semi-mangrove plant *Myoporum bontioides*. A. Gray, which led to the isolation of two new farnesane sesquiterpene alkaloids, myoporumines A (**351**) and B (**352**) (Figure 9), which displayed potent anti-MRSA activity with MIC value of 6.25 µg/mL [83]. Two aromadendrane sesquiterpene 1-isothiocyanatoaromadendrane (**353**) and **347**, one spioaxane-type sesquiterpenoid axamide-3 (**354**), and two pupukeanane-type sesquiterpenoids (**349**, **350**) (Figure 9), were isolated from the nudibranchs *Phyllidiella pustulosa* and *Phyllidia coelestis* [67].

### 2.8. Tremulane, Daucane, Brasilane, Salvialane, Aristolane, Bergamotane and Valerane Sesquiterpenoids

**Figure 10:**
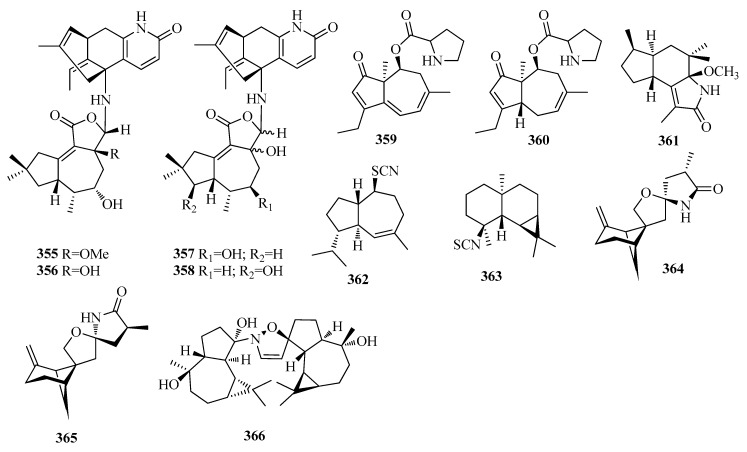
The structures of compounds **355**–**366**.

Huptremules A–D (compounds **355**–**358**) (Figure 10) featuring unusual sesquiterpenoid-alkaloid hybrid structures that integrate the characteristics offungal metabolites (tremulane sesquiterpenoids) and the exogenous substrate, were isolated from a fungal endophyte of *Huperzia serrata* [84]. Compound **355**–**358** selectively inhibited acetylcholinesterase activities, with IC_50_ values of 0.99, 2.17, 0.11 and 0.06 μM, respectively [84]. Two daucane-type sesquiterpenoids, aculeneA (**359**) and B (**360**) (Figure 10), were identified from *Aspergillus aculeatus*, which were tested for antifungal activity against *Candida albicans*. However, all showed only weak orno activity [85]. One brasilane-type sesquiterpenoid, named diaporol L (**361**) (Figure 10) was isolated from *Diaporthe* sp., an endophytic fungus associated with the leaves of *Rhizophora stylosa* collected in Hainan Province, China [86]. One salvialane-type sesquiterpene halichon E (**362**) and one aristolane sesquiterpene epipolasin A (**363**) (Figure 10) were obtained from the Thai sponge *Halichondria* sp. [65]. Sporulaminals A (**364**) and B (**365**) (Figure 10), a pair of unusual epimericspiroaminal derivatives, bearing 6/4/5/5 tetracyclic ring system derived from bergamotane sesquiterpenoid, were isolated from a marine-derived fungus Paraconiothyrium sporulosum YK-03 [87]. Volvalerine A (**366**) (Figure 10), a novel *N*-containing valerane bisesquiterpenoid derivative with a dihydroisoxazole ring, was isolated from the roots of *Valeriana officinalis* var. *latifolia* [88]. Compound **366** was also evaluated for their enhancing activity on NGF mediated neurite outgrowth in PC12 cells. The result indicatedthat the proportion of the NGF-induced neurite-bearing cells (with NGF 5 ng/mL) was not enhanced by compound **366** at 50 μM [88].

### 2.9. Cyclonerane, Axane, Nardosinane, Zizaane, Eremophilane, and Guaiane Sesquiterpenoids

**Figure 11:**
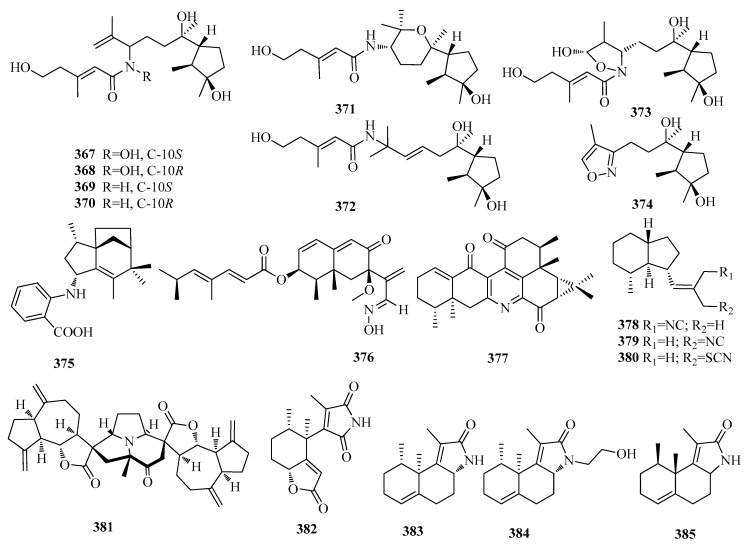
The structures of compounds **367**–**385**.

The nitrogenous cycloneranesesquiterpenescyclonerin A (**367**) and B (**368**) along with seven new congeners―deoxycyclonerins A–D (**369**–**372**), cyclonerinal (**373**), and cyclonerizole (**374**) (Figure 11)―were isolated from the culture of a marine algicolous strain(A-YMD-9-2) of *Trichoderma asperellum* [89]. And, compounds (**367**–**374**) showed significant cytotoxic activityagainst harmful microalgae Chattonella marina with the IC_50_ value of 2.1–30 μg/mL [89]. Antartin (**375**) (Figure 11), a cytotoxic zizaane-type sesquiterpenoid was obtained from a *Streptomyces* sp. SCO736, isolated from an Antarcticmarine sediment, and showed cytotoxicity against A549, H1299, and U87 cancer cell lines by causing cell cycle arrest at the G1 phase [90]. One eremophilane sesquiterpene dendryphiellin J (**376**) (Figure 11) was isolated from the marine-derived fungus *Cochliobolus lunatus* SCSIO41401 [91]. Compound **376**, a rare naturally occurring aldoxime analogue, displayed cytotoxicities against ACHN and HepG-2 cells with IC_50_ values of 3.1 and 5.9 μM, respectively [91]. One unusual sesquiterpenoid dimer, nardochinoid B (**377**) (Figure 11) was isolated from *Nardostachys chinensis* Batal [92]. Compound **377** is the first nitrogen-containing nornardosinane-aristolane sesquiterpene conjugate. The ED_50_ of compound **377** on the production of NO was 5.73, and obviously inhibited LPS-inducediNOS and COX-2 protein expression in a dose-dependent way, and increased HO-1 protein expression at the concentration of 10 μM [92].Three axane sesquiterpenoid isonitrile pictaisonitrile-1 (**378**), pictaisonitrile-2 (**379**), and cavernothiocyanate (**380**) (Figure 11) were extracted from *hyllidiapicta* collected from Bali, Indonesia [71]. Vlasoulamine A (**381**) (Figure 11), an unprecedented guaiane sesquiterpene lactone dimerfeaturing a fully hydrogenated pyrrolo[2,1,5-*cd*] indolizine core, was isolated from the roots of *Vladimiria souliei* [93]. Moreover, **381** exhibited neuroprotective activity whenevaluated for glutamate-induced cytotoxicity, nuclear Hoechst 33,258 staining, and measuring intracellular reactive oxygen species levels, using a rat pheochromocytoma PC12 cell-based model system [93]. Clavukoellians A–D (**382**-**385**) (Figure 11), highly rearranged nardosinane Sesquiterpenoids with antiangiogenic activity were purified from the marine soft coral *Clavularia koellikeri* [94]. Compound **382** has aunique skeleton with both lactone and maleimide ring systems, which is rare in natural products, and appears to be formed byoxidative cleavage of the C-7/C-8 bond of a nardosinane precursor with inhibiting the migration of the human umbilical veinendothelial cells (HUVECs) at 2.5 μM [94].

### 2.10. Others

**Figure 12:**
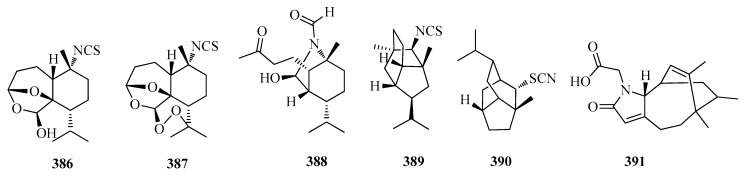
The structures of compounds **386**–**391**.

Five sesquiterpene isocyanides, isothiocyanates, thiocyanates, andformamides―halichon A (**386**), halichon B (**387**), halichon F (**388**), halichon H (**389**), and (+)-2-thiocyanatoneopupukeanane (**390**) (Figure 12) ― were isolated from the Thai sponge *Halichondria* sp. [65]. Lamellodysidine B (**391**) (Figure 12), a sesquiterpenes isolated from the marine sponge *Lamellodysidea herbacea*, collected inIndonesia [95]. Biological activities of **391** was tested in our in-house screening including cytotoxicity, antimicrobial activities, inhibitory activity of the cholesterol ester accumulation in macrophages, inhibitory activity of the RANKL-induced formation of multinuclear osteoclasts, and inhibitory activities of the ubiquitin-proteasome system (proteasome, E1,Ubc13 (E2)−Uev1A interaction, p53-Mdm2 (E3) interaction, and USP7). However, no significant activity was detected forthe compound [95].

## 3. Occurrence

Natural nitrogenous sesquiterpenoids are mainly distributed in species of plants belonging to the Celastraceae, Saxifragaceae, Zingiberaceae, Asteraceae, Burseraceae, Phyllanthaceae, Magnoliaceae, Lamiaceae, Myoporaceae, and Valerianaceae families, marine sponges belonging to the Dysiseidae, Thorectidae, Spongiidae, and Halichodriae families, soft corals belonging to the Xeniidae and Clavulariidae families, phyllidid nudibranchs belonging to the Phyllidiidae family, marine slugs belonging to the Dotidae family), fungi belonging to the Trichocomaceae, Eurotiaceae, Parmulariaceae, Phanerochaetaceae, Diaporthaceae, and Pezizaceae families, bacteria belonging to the Pseudomonadaceae family, and actinomyces belonging to the Streptomycetaceae family (Table 3). Dihydroagarofuran sesquiterpenoids have been isolated from the roots of *Maytenus mekongensis*, the stems of *M. oblongata*, the leaves of *M. spinosa*, the roots and leaves of *Tripterygium wilfordii*, the stems of *T. regelii*, the root barks of *T. hypoglaucum*, the fruits of *Celastrus orbiculatus*, the seeds of *C. paniculatus*, the root barks of *C. angulatus*, the stems of *Euonymus alatus*, the whole plants of *Parnassia wightiana*, the leaves of *Monimopetalum chinense*. Friedo-drimane and drimane sesquiterpenes have been extracted from maring sponges of the following species: *Dysidea* sp., *D. avara*, *D. fragilis*, *D. cinerea*, *D. septosa*, *Dactylospongia* sp., *D. elegans*, and *D. metachromia*. Drimane sesquiterpenoids have been purified from the fungi *Aspergillus ochraceus*, *A. aculeatus*, *Talaromyces* minioluteus, and *Penicillium* sp. ZZ1283, the bacterium *Saccharomonospora* sp. CNQ-490, and the actinomycete *Streptomyces* sp. Eudesmane sesquiterpenoids have been identified inmarine sponges of *Halichondria* sp., *H. okadai*, *Axinyssa* sp., and *A. variabilis*, the soft coral *Cespitularia taeniata*, phyllidid nudibranchs of the *Phyllidiella* sp., *P. pustulosa*, and *P. ocellate* species and the plants *Curcuma phaeocaulis* and *Inula helenium* L. Germacranese squiterpenoids were isolated from the plants *Onopordum alexandrinum*, *Magnolia kobus*, and *Salvia scapiformis*. Cadinane sesquiterpenes were extracted from the plant *Resina commiphora*, marine sponges like *Halichondria* sp. and *Axinyssa* sp., phyllidid nudibranchs of the *Phyllidiella* sp. Bisabolane sesquiterpenoids have been isolated from *Phyllanthus acidus* (L.) skeels, *Halichondria* sp. *Phyllidiella* sp., *Paraconiothynium brasiliense* and *P. sporulosum*.

## 4. Conclusions

In summary, a total of 391 bioactive nitrogenous sesquiterpenoids have been isolated and characterized from plants, microorganisms, and marine organisms at the past ten years. This report systematically describes the occurrence, isolation, structures and biological activities ofthese nearly 400 natural products that contain a nitrogen-carbon/nitrogen-nitrogen/nitrogen-sulfurbond. These natural products are dispersed over severalstructural classes, isolated from many different sources (bothmarine and terrestrial) and possess a diverse array of biological activities. It can be concluded that the structure types are obviously related to the species sources, and the bioactivities of nitrogenous sesquiterpenoids are obviously related to structure types, being particularly important their cytotoxic activities. The important points arising from this review are the following: (1) There are few structural types of *N*-containing sesquiterpenes in plants, while the structural types of sesquiterpenes with nitrogen in marine resources and microorganisms are various and diverse. (2) Dihydroagarofuran sesquiterpenoids were considered the most widespread and characteristic metabolites of the plants of Celastraceae, which are well recognized as characteristic metabolitesand important chemotaxonomic markers or indicators of the family, exceptforsome β-dihydroagarofurans obtained from the Saxifragaceae species *Parnassia wightiana*. (3) Sponges and their associated microorganisms are the largest contributors of nitrogenous sesquiterpenoids. Rearranged 4,9-friedo-drimaneterpenoid skeletons represent the majority ofnitrogen-contenting sesquiterpenes isolated from marine sponges. The types of sesquiterpenoids that are the most abundant among the marine organisms, *Halichondria* sp. (sponge) and *Phyllidiella* sp. (nudibranchs), are all sesquiterpene isocyanides, isothiocyanates, thiocyanates, and formamides. (4) Nitrogenous sesquiterpenes are rich in microorganisms, such as fungus, bacteria and actinomyces and the main skeleton types are drimane, bisabolane, farnesane, tremulane sesquiterpenoids and so on. (5) Dihydroagarofuran sesquiterpenoids show significant anti-inflammatory, neuroprotective, and immunosuppressive effects, while sesquiterpenes isolated from marine organisms exhibit remarkable antitumor cytotoxic activities. Due to the rich activities and structural diversity of N-contenting sesquiterpenes, researchers have not stopped exploring and studying such compounds. We hope this review will stimulate further researchinto this interesting class of nitrogenous secondary metabolites.

## Figures and Tables

**Table 1:** Reported structures ofdihydroagarofuran sesquiterpenoids **1**–**144**.

No	Name	R_1_	R_2_	R_3_	R_4_	R_5_	R_6_	R_7_	R_8_	R_9_	Type	Ref
**1**	Mekongensine	OAc	OBz	βOAc	βOAc	OAc	OH	βOAc	OAc	H	A	[17]
**2**	7-*epi*-Mekongensine	OAc	OBz	αOAc	βOAc	OAc	OH	βOAc	OAc	H	A	[17]
**3**	1-*O*-Benzoyl-1-deacetylmekongensine	OBz	OBz	βOAc	βOAc	OAc	OH	βOAc	OAc	H	A	[17]
**4**	9′-Deacetoxymekongensine	OAc	OBz	βOAc	βOAc	OAc	OH	βOAc	H	H	A	[17]
**5**	1-*O*-Benzoyl-1-deacetyl-9′-deacetoxymekongensine	OBz	OBz	βOAc	βOAc	OAc	OH	βOAc	H	H	A	[17]
**6**	7-*epi*-Euojaponine A	OBz	OH	αOAc	βOAc	OAc	OH	βOAc	H	CH_3_	B	[17]
**7**	2-*O*-Benzoyl-2-deacetylmayteine	OBz	OAc	βOAc	βOAc	OAc	OH	βOBz	H	CH_3_	B	[17,24]
**8**	7-*epi*-5-*O*-Benzoyl-5-deacetylperitassine A	OAc	OBz	αOAc	βOAc	OAc	OH	βOAc	H	CH_3_	C	[17]
**9**	7-*epi*-Euonymine	OAc	OAc	αOAc	βOAc	OAc	OH	βOAc	H	CH_3_	B	[17]
**10**	Mayteine	OBz	OAc	βOAc	βOAc	OAc	OH	βOAc	H	CH_3_	B	[17]
**11**	7-*epi*-Mayteine	OBz	OAc	αOAc	βOAc	OAc	OH	βOAc	H	CH_3_	B	[17]
**12**	Euonymine	OAc	OAc	βOAc	βOAc	OAc	OH	βOAc	H	CH_3_	B	[17,18,21,26]
**13**	9′-*O*-Acetyl-7-deacetoxy-7-oxowilfortrine	OAc	OAc	O	βOAc	OAc	OH	βOFu	OAc	H	A	[18,26]
**14**	9′-*O*-Acetylwilfortrine	OAc	OAc	βOAc	βOAc	OAc	OH	βOFu	OAc	H	A	[18]
**15**	9′-*O*-Furanoylwilfordine	OAc	OAc	βOAc	βOAc	OAc	OH	βOBz	OFu	H	A	[18]
**16**	7-*O*-Benzoyl-5,7-dideacetylwilformine	OAc	OH	βOBz	βOAc	OAc	OH	βOAc	H	H	A	[18]
**17**	Wilfortrine	OAc	OAc	βOAc	βOAc	OAc	OH	βOFu	OH	H	A	[18,21,26]
**18**	Wilforgine	OAc	OAc	βOAc	βOAc	OAc	OH	βOFu	H	H	A	[18,23,26]
**19**	Wilfordine	OAc	OAc	βOAc	βOAc	OAc	OH	βOBz	OH	H	A	[18,26]
**20**	Wilforine	OAc	OAc	βOAc	βOAc	OAc	OH	βOBz	H	H	A	[18,26]
**21**	Wilformine	OAc	OAc	βOAc	βOAc	OAc	OH	βOAc	H	H	A	[18]
**22**	Wilforidine	OAc	OAc	βOAc	βOAc	OAc	OH	βOH	OH	H	A	[18]
**23**	Cangorinine E-1	OAc	OBz	βOAc	βOAc	OAc	OH	βOAc	H	CH_3_	B	[18]
**24**	Ebenifoline E-II	OBz	OBz	βOAc	βOAc	OAc	OH	βOAc	H	CH_3_	B	[18]
**25**	Neoeuonymine	OAc	OH	βOAc	βOAc	OAc	OH	βOAc	H	CH_3_	B	[18,24,26]
**26**	Peritassine A	OAc	OAc	βOAc	βOAc	OAc	OH	βOAc	H	CH_3_	C	[18,21,31]
**27**	Wilfornine G	OAc	OAc	βONic	βOAc	OAc	OH	βOAc	H	CH_3_	C	[18]
**28**	Regelidine	OBz	ONic	H	αOBz	H	OH	H	H	H	D	[18,24,28]
**29**	9-*O*-*trans*-Cinnamoyl-9-debenzoylregelidine	OBz	ONic	H	αO*t*Cin	H	OH	H	H	H	D	[18]
**30**	1β-Acetoxy-8α,9β-dibenzoyloxy-13-nicotinoyloxy-β-dihydroagarofuran	OAc	H	αOBz	βOBz	ONic	H	H	H	H	D	[19]
**31**	1β,2β-Diacetoxy-9α-benzoyloxy-13-nicotinoyloxy-β-dihydroagarofuran	OAc	H	H	αOBz	ONic	H	βOAc	H	H	D	[19]
**32**	Hypoglaunine E	OAc	OH	βOAc	βOAc	OFu	OH	βOAc	OH	CH_3_	C	[20,21,31]
**33**	Hypoglaunine F	OAc	OH	βOAc	βOAc	OAc	OH	βOFu	OH	CH_3_	C	[20,31]
**34**	Triptersinine A	O*t*Cin	OH	O	βONic	OAc	OH	H	H	H	D	[21]
**35**	Triptersinine B	O*c*Cin	OH	O	βONic	OAc	OH	H	H	H	D	[21]
**36**	Triptersinine C	βO*t*Cin	OH	βOAc	βONic	OAc	OH	H	H	H	D	[21]
**37**	Triptersinine D	O*c*Cin	OH	βOAc	βONic	OAc	OH	H	H	H	D	[21]
**38**	Triptersinine E	O*c*Cin	OAc	βOAc	βONic	OAc	OH	H	H	H	D	[21]
**39**	Triptersinine F	OAc	ONic	βOAc	βOFu	OAc	OH	H	H	H	D	[21]
**40**	Triptersinine G	OAc	OAc	βONic	βOFu	OAc	OH	H	H	H	D	[21]
**41**	Triptersinine H	OFu	OAc	βONic	βOFu	OAc	OH	H	H	H	D	[21]
**42**	Triptersinine L	OAc	ONic	βOAc	αOTig	OAc	OH	H	H	H	D	[21]
**43**	Wilfordinine A	OAc	OAc	βOAc	βOAc	OAc	OH	βOH	H	CH_3_	C	[21,31]
**44**	Hypoglaunine A	OAc	OAc	βOAc	βOAc	OFu	OH	βOAc	OH	CH_3_	C	[21,31]
**45**	Wilfordinine E	OAc	OAc	βOAc	βOAc	OAc	OH	βOAc	H	H	F	[21]
**46**	Euonine	OAc	OAc	βOAc	βOAc	OAc	OH	βOAc	H	H	A	[21,31]
**47**	Evonine	OAc	OAc	O	αOAc	OAc	OH	αOAc	H	CH_3_	G	[22]
**48**	Neoevonine	OAc	OH	O	αOAc	OAc	OH	αOAc	H	CH_3_	G	[22]
**49**	1β,2β,5α,8β,11-Pentaacetoxy-4α-hydroxy-3α-(2-methylbutanoyl)-15-nicotinoyl-7-oxo-dihydroagarofuran	OAc	OAc	O	αOAc	OAc	OH	αOAc	OMeBu	ONic	E	[22]
**50**	Triptersinine M	O*t*Cin	OAc	βOAc	βONic	OAc	OH	H	H	H	D	[23]
**51**	Triptersinine N	ONic	OFu	βOAc	βOFu	OAc	OH	H	H	H	D	[23]
**52**	Triptersinine O	OFu	OFu	βOAc	βONic	OAc	OH	H	H	H	D	[23]
**53**	Triptersinine P	OTig	OAc	βONic	βONic	OAc	OH	H	H	H	D	[23]
**54**	Triptersinine Q	OFu	OAc	βONic	βOTig	OAc	OH	H	H	H	D	[23]
**55**	Triptersinine R	OAc	OAc	βONic	αOFu	OAc	OH	H	H	H	D	[23]
**56**	Triptersinine S	OAc	OFu	βOAc	βONic	OAc	OH	H	H	H	D	[23]
**57**	Triptersinine T	OAc	OH	βOAc	βONic	OAc	H	H	H	H	D	[23]
**58**	Tripterygiumine A	OAc	OAc	-	βOAc	-	OH	βOBz	H	CH_3_	H	[24]
**59**	Tripterygiumine B	OAc	OAc	βOBz	βOAc	OAc	OH	βOAc	H	CH_3_	B	[24]
**60**	Tripterygiumine C	OAc	OBz	βOAc	βOAc	OAc	OH	βOBz	H	CH_3_	B	[24]
**61**	Tripterygiumine D	OH	OBz	βOH	βOH	OH	OH	βOH	H	CH_3_	B	[24]
**62**	Tripterygiumine E	OAc	OH	βOAc	βOAc	OAc	OH	βOFu	H	CH_3_	B	[24]
**63**	Tripterygiumine F	OAc	OFu	βOAc	βOAc	OAc	OH	βOBz	H	CH_3_	B	[24]
**64**	Tripterygiumine G	OAc	OBz	βOAc	βOAc	OAc	OH	βOFu	H	CH_3_	B	[24]
**65**	Tripterygiumine H	OH	OAc	βOH	βOH	OH	OH	βOH	H	CH_3_	B	[24]
**66**	Tripterygiumine I	OAc	OH	βOAc	βOAc	OAc	OH	βOBz	H	CH_3_	B	[24]
**67**	Tripterygiumine J	OAc	OH	βOH	βOAc	OAc	OH	βOAc	H	CH_3_	B	[24]
**68**	Tripterygiumine K	OAc	OH	βOAc	βOAc	OBz	OH	βOH	H	CH_3_	B	[24]
**69**	Tripterygiumine L	ONic	OH	βOAc	βOAc	OAc	OH	βOAc	H	CH_3_	B	[24]
**70**	Hyponine D	OAc	OBz	βOAc	βOAc	OAc	OH	βONic	H	CH_3_	B	[24]
**71**	Hexadesacetyleuomynine	OH	OH	βOH	βOH	OH	OH	βOH	H	CH_3_	B	[24]
**72**	Euojaponine A	OBz	OH	βOAc	βOAc	OAc	OH	βOAc	H	CH_3_	B	[24]
**73**	Hyponine C	OAc	OAc	βOAc	βOAc	OBz	OH	βOAc	H	CH_3_	B	[24]
**74**	7-Acetyloxy-*O*^11^-benzoyl-*O*^2,11^- deacetyl-7- deoxoevonine	OAc	OAc	βOAc	βOAc	OBz	OH	βOH	H	CH_3_	B	[24]
**75**	4-Hydroxy-7-*epi*-chuchuhuanine E-V	OAc	OAc	βOAc	βOAc	OAc	OH	βOH	H	CH_3_	B	[24,26]
**76**	Wilfornine F	OAc	OBz	βOAc	βOAc	OAc	OH	βOH	H	CH_3_	B	[24]
**77**	Tripterygiumine M	OAc	OH	O	βOAc	OAc	OH	βOBz	H	H	A	[24]
**78**	Tripterygiumine N	OAc	OH	O	βOAc	OAc	OH	βOBz	OFu	H	A	[24]
**79**	Tripterygiumine O	OAc	OH	βOAc	βOAc	OAc	OH	βOFu	OBz	H	A	[24]
**80**	Tripterygiumine P	OH	OAc	βOH	βOH	OH	OH	βOH	OBz	H	A	[24]
**81**	Tripterygiumine Q	OH	OAc	βOH	βOH	OH	OH	βOH	OFu	H	A	[24]
**82**	Triptonine B	OAc	OAc	βOAc	βOAc	OAc	OH	βOFu	OFu	H	A	[24]
**83**	1-Desacetylwilforgine	OH	OAc	βOAc	βOAc	OAc	OH	βOFu	H	H	A	[24]
**84**	Alatamine	OAc	OAc	O	βOAc	OAc	OH	βOBz	OH	H	A	[24]
**85**	Alatusinine	OAc	OAc	βOAc	βOAc	OAc	OH	βOAc	OH	H	A	[24]
**86**	Wilforzine	OAc	OH	βOAc	βOAc	OAc	OH	βOBz	H	H	A	[24]
**87**	Wilforjine	OAc	OAc	βOAc	βOAc	OAc	OH	βOH	H	H	A	[24,26]
**88**	Tripterygiumine R	ONic	OH	H	αOBz	H	OH	H	H	H	D	[24]
**89**	1β,5α,11-Triacetoxy-7β-benzoyl-4α-hydroxy-8β- nicotinoyl-dihydroagarofuran	OAc	OAc	βOBz	αONic	OAc	OH	H	H	H	D	[24]
**90**	Wilforcidine	OBz	ONic	H	αOtCin	H	OH	H	H	H	D	[24]
**91**	5α-Benzoyl-4α-hydroxy-1β,8α-dinicotinoyl-dihydroagarofuran	ONic	OBz	H	αONic	H	OH	H	H	H	D	[24]
**92**	1α,2α,6β,8β,9α,15-Hexacetoxy-4β-hydroxy-3β,13-[2′-(3-carboxybutyl)]nicotinic acid-dicarbolactone-β-di hydroagarofuran	OAc	OAc	βOAc	αOAc	OAc	OH	αOAc	H	H	I	[25]
**93**	1α,2α,9α,15-Tetracetoxy-4β,6β-dihydroxy-8-oxo,3β,13-[4′-(3-carboxybutyl)]nicotinicacid-dicarbolactone- β-dihydroagarofuran	OAc	OH	O	βOAc	OAc	OH	αOAc	H	H	J	[25]
**94**	1α,2α,9α,15-Tetracetoxy-4β,6β,8β-trihydroxy-3β,13-[4′-(3-carboxybutyl)]nicotinic acid-dicarbolactone- β-dihydroagarofuran	OAc	OH	βOH	βOAc	OAc	OH	αOAc	H	H	J	[25]
**95**	1α,2α,8β,9α,15-Pentacetoxy-4β,6β-dihydroxy-3β,13-[4′-(3-carboxybutyl)]nicotinic acid-dicarbolactone-β- dihydroagarofuran	OAc	OH	βOAc	βOAc	OAc	OH	αOAc	H	H	J	[25]
**96**	Tripterygiumine S	OAc	OAc	O	βOAc	OAc	OH	βOH	OFu	H	A	[26]
**97**	Tripterygiumine T	OAc	OH	O	βOAc	OAc	OH	βOH	OH	H	A	[26]
**98**	Tripterygiumine U	OAc	OAc	O	βOAc	OAc	OH	βOH	H	H	A	[26]
**99**	Tripterygiumine V	OAc	OAc	βOAc	βOAc	OAc	OH	βOH	OBz	H	A	[26]
**100**	Tripterygiumine W	OFu	OBz	βOAc	βOAc	OAc	OH	βOH	H	CH_3_	B	[26]
**101**	Wilfornine A	OAc	OAc	βOAc	βOAc	OAc	OH	βOAc	OBz	H	A	[26]
**102**	Wilfornine D	OAc	OAc	βOAc	βOAc	OAc	OH	βOAc	OFu	H	A	[26]
**103**	Tripfordine A	OAc	OAc	βOAc	βOAc	OAc	OH	βOH	OH	H	A	[26]
**104**	2-Debenzoyl-2-nicotinoylwilforine	OAc	OAc	βOAc	βOAc	OAc	OH	βONic	H	H	A	[26]
**105**	(+)-(1*R*,2*S*,4*S*,5*S*,6*R*,7*R*,9*S*,10*R*)-1,2,15-Triacetoxy-9-benzoyloxy-6-nicotinoyloxydihydro-β-agarofuran	OAc	ONic	H	βOBz	OAc	OH	αOAc	H	H	E	[27]
**106**	Triptregeline A	ONic	OH	βOAc	αOBz	OAc	OH	αOAc	H	H	E	[28]
**107**	Triptregeline B	ONic	OAc	αOAc	αOBz	OAc	OH	H	H	H	E	[28]
**108**	Triptregeline C	ONic	OAc	αOH	αOBz	OH	OH	H	H	H	E	[28]
**109**	Triptregeline D	OFu	OAc	αONic	αOBz	OAc	OH	H	H	H	E	[28]
**110**	Triptregeline E	OFu	OH	αONic	αOBz	OAc	OH	H	H	H	E	[28]
**111**	Triptregeline F	OAc	OH	αONic	αOBz	OAc	OH	H	H	H	E	[28]
**112**	Triptregeline G	OFu	OH	αONic	αOAc	OAc	OH	H	H	H	E	[28]
**113**	Triptregeline H	OBz	OAc	αOH	αONic	OAc	OH	H	H	H	E	[28]
**114**	Triptregeline I	OFu	ONic	H	βOBz	H	OH	βOAc	H	H	E	[28]
**115**	Triptregeline J	OBz	ONic	H	βOBz	H	OH	H	H	H	E	[28]
**116**	1α, 6β, 15-Triacetoxy-8α-benzoyloxy-4β-hydroxyl -9α-(3-nicotinoyloxy)-dihydro-β-agarofuran	OAc	OAc	αOBz	αONic	OAc	OH	H	H	H	E	[28]
**117**	Dimacroregeline A	OH	OAc	H	αOH	-	OH	αOH	H	CH_3_	K	[29]
**118**	Dimacroregeline B	OH	OAc	OAc	αOH	-	OH	αOH	H	CH_3_	K	[29]
**119**	Triptonine A	OAc	OAc	-	αOAc	-	OH	αOAc	H	CH_3_	L	[29]
**120**	4-Deoxyalatamine	OAc	OAc	O	αOAc	OAc	H	αOAc	OH	H	I	[30]
**121**	1-*O*-Benzoyl-1-deacetyl-4-deoxyalatamine	OBz	OAc	O	αOAc	OAc	H	αOAc	OH	H	I	[30]
**122**	1, 2-*O*-Dibenzoyl-1, 2-deacetyl-4-deoxyalatamine	OBz	OAc	O	αOAc	OAc	H	αOBz	OH	H	I	[30]
**123**	4-Deoxyisowilfordine	OAc	OAc	βOAc	αOAc	OAc	H	αOBz	OH	H	J	[30]
**124**	Triptersinine U	OAc	OAc	βOAc	βOAc	OAc	OH	βOAc	αONic	ONic	D	[31]
**125**	Hypoglaunine B	OAc	OAc	βOAc	βOAc	OFu	OH	βOAc	OH	CH_3_	C	[31]
**126**	Triptersinine Z4	OFu	OAc	βOAc	βONic	OAc	H	H	H	H	D	[32]
**127**	Triptersinine Z5	OAc	OFu	βOAc	βONic	OAc	H	H	H	H	D	[32]
**128**	Triptersinine Z6	OFu	OFu	βOAc	βONic	OAc	H	H	H	H	D	[32]
**129**	Triptersinine Z7	O*c*Cin	OAc	βOAc	βONic	OAc	H	H	H	H	D	[32]
**130**	Triptersinine Z8	O*t*Cin	OAc	βOAc	βONic	OAc	H	H	H	H	D	[32]
**131**	Euojaponine C	OBz	OBz	βOAc	βOAc	OAc	OH	βOH	H	CH_3_	B	[32]
**132**	Triptersinine Z9	O*c*Cin	OFu	βOAc	βONic	OAc	OH	H	H	H	D	[33]
**133**	Triptersinine Z10	O*t*Cin	OFu	βOAc	βONic	OAc	OH	H	H	H	D	[33]
**134**	Triptersinine Z11	O*t*Cin	OAc	βONic	βOFu	OAc	OH	H	H	H	D	[33]
**135**	Triptersinine Z12	O*c*Cin	OAc	βONic	βOFu	OAc	OH	H	H	H	D	[33]
**136**	Triptersinine Z13	ONic	OFu	βOAc	βOTig	OAc	OH	H	H	H	D	[33]
**137**	Triptersinine Z14	OAc	OFu	βONic	βOTig	OAc	OH	H	H	H	D	[33]
**138**	Chinese bittersweet alkaloid A	OAc	OAc	βOAc	βOAc	OiBu	OH	βOH	H	CH_3_	B	[34]
**139**	Chinese bittersweet alkaloid B	OAc	OAc	βOAc	βOAc	OiBu	OH	βOAc	H	CH_3_	B	[34]
**140**	Monimin I	ONic	ONic	H	αOAc	H	H	H	H	H	E	[35]
**141**	Monimin II	ONic	ONic	αOH	αOBz	H	H	H	H	H	E	[35]
**142**	Tripteryford C	ONic	OH	βOAc	αOAc	OAc	H	αOAc	βOH	H	E	[36]
**143**	Tripteryford E	ONic	OAc	αOH	βOFu	OAc	OH	αOAc	βOH	H	E	[36]
**144**	Celaspaculin G	OAc	OBz	βOAc	αONic	H	OH	H	H	H	E	[37]

**Table 2:** Reported structures offriedo-drimane sesquiterpenoids.

No	Name	R_1_	R_2_	R_3_	R_4_	R_5_	R_6_	R_7_	Type	Ref
**161**	18-Aminoarenarone	H	NH_2_	H	αH	αCH_3_	βCH_3_	βCH_3_	I	[43]
**162**	19-Aminoarenarone	NH_2_	H	H	αH	αCH_3_	βCH_3_	βCH_3_	I	[43]
**163**	18-Methylaminoarenarone	H	NHCH_3_	H	αH	αCH_3_	βCH_3_	βCH_3_	I	[43]
**164**	19-Methylaminoarenarone	NHCH_3_	H	H	αH	αCH_3_	βCH_3_	βCH_3_	I	[43]
**167**	Nkijinol B	OH	OH	H	H	βCH_3_	βCH_3_	βCH_3_	II	[44]
**168**	Smenospongine B	H	NHCH_2_COOH	OH	αH	βCH_3_	βCH_3_	βCH_3_	I	[44]
**169**	Smenospongine C	H	NH(CH_2_)_2_COOH	OH	H	βCH_3_	βCH_3_	βCH_3_	II	[44]
**170**	Nakijinol B diacetate	OAc	OAc	H	αH	βCH_3_	βCH_3_	βCH_3_	I	[44]
**171**	(−)-3′-Methylaminoavarone	H	NHCH_3_	H	αH	βCH_3_	βCH_3_	βCH_3_	III	[45]
**172**	(−)-4′-Methylamino-avarone	NHCH_3_	H	H	αH	βCH_3_	βCH_3_	βCH_3_	III	[45]
**173**	(−)-*N*-Methylmelemeleone-A	H	N(CH_3_)(CH_2_)_2_SO_3_H	H	αH	βCH_3_	βCH_3_	βCH_3_	III	[45]
**174**	Smenospongine	H	NH_2_	OH	αH	βCH_3_	βCH_3_	βCH_3_	IV	[46,50,52]
**175**	Glycinylilimaquinone	H	NHCH_2_COOH	OH	αH	βCH_3_	βCH_3_	βCH_3_	IV	[46]
**176**	5-*epi*-Nakijiquinone S	H	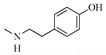	OH	αH	αCH_3_	βCH_3_	βCH_3_	V	[47]
**177**	5-*epi*-Nakijiquinone Q	H	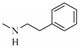	OH	αH	αCH_3_	βCH_3_	βCH_3_	V	[47]
**178**	5-*epi*-Nakijiquinone T	H	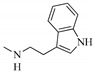	OH	αH	αCH_3_	βCH_3_	βCH_3_	V	[47]
**179**	5-*epi*-Nakijiquinone U	H	NH(CH_2_)_3_SCH_3_	OH	αH	αCH_3_	βCH_3_	βCH_3_	V	[47]
**180**	5-*epi*-Nakijiquinone N	H	NH(CH_2_)_2_CH(CH_3_)_2_	OH	αH	αCH_3_	βCH_3_	βCH_3_	V	[47]
**181**	5-*epi*-Nakijinol C	OH	OCH_3_	CH_3_	αH	αCH_3_	βCH_3_	βCH_3_	VI	[47]
**182**	5-*epi*-Nakijinol D	CH_3_	CH_3_	-	αH	αCH_3_	βCH_3_	βCH_3_	VII	[47]
**183**	Dysidaminone A	NHCH_2_CH(CH_3_)_2_	H	H	αH	βCH_3_	βCH_3_	βCH_3_	III	[48]
**184**	Dysidaminone B	NHCH_2_CH(CH_3_)CH_2_CH_3_	H	H	αH	βCH_3_	βCH_3_	βCH_3_	III	[48]
**185**	Dysidaminone C	H	N(CH_3_)_2_	H	αH	βCH_3_	βCH_3_	βCH_3_	III	[48]
**186**	Dysidaminone D	N(CH_3_)_2_	H	H	αH	βCH_3_	βCH_3_	βCH_3_	III	[48]
**187**	Dysidaminone E	H	NHCH_2_CH(CH_3_)_2_	H	αH	βCH_3_	βCH_3_	βCH_3_	III	[48]
**188**	Dysidaminone F	H	NHCH_2_CH(CH_3_)CH_2_CH_3_	H	αH	βCH_3_	βCH_3_	βCH_3_	III	[48]
**189**	Dysidaminone G	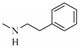	H	H	αH	βCH_3_	βCH_3_	βCH_3_	III	[48]
**190**	Dysidaminone H	H	NHCH_3_	H	αH	βCH_3_	βCH_3_	βCH_3_	I	[48]
**191**	Dysidaminone I	NHCH_3_	H	H	αH	βCH_3_	βCH_3_	βCH_3_	I	[48]
**192**	Dysidaminone J	H	N(CH_3_)_2_	H	αH	βCH_3_	βCH_3_	βCH_3_	I	[48]
**193**	Dysidaminone K	NHCH_2_CH(CH_3_)2	H	H	αH	βCH_3_	βCH_3_	βCH_3_	I	[48]
**194**	Dysidaminone L	NHCH_2_CH(CH_3_)CH_2_CH_3_	H	H	αH	βCH_3_	βCH_3_	βCH_3_	I	[48]
**195**	Dysidaminone M	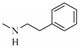	H	H	αH	βCH_3_	βCH_3_	βCH_3_	I	[48]
**196**	18-Methylaminoavarone	H	NHCH_3_	H	αH	βCH_3_	βCH_3_	βCH_3_	III	[48]
**197**	19-Methylaminoavarone	NHCH_3_	H	H	αH	βCH_3_	βCH_3_	βCH_3_	III	[48]
**198**	18-Aminoavarone	H	NH_2_	H	αH	βCH_3_	βCH_3_	βCH_3_	III	[48]
**199**	19-Aminoavarone	NH_2_	H	H	αH	βCH_3_	βCH_3_	βCH_3_	III	[48]
**200**	18-Phenethylaminoavarone	H	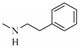	H	αH	βCH_3_	βCH_3_	βCH_3_	III	[48]
**201**	Popolohuanone D	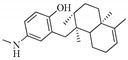	H	H	αH	βCH_3_	βCH_3_	βCH_3_	III	[48]
**202**	(-)-Nakijinol E	OH	OCH_3_	H	CH_3_	βCH_3_	βCH_3_	βCH_3_	II	[49]
**203**	(+)-5-*epi*-Nakijinol E	OH	OCH_3_	H	CH_3_	αCH_3_	βCH_3_	βCH_3_	II	[49]
**204**	Nakijinone A	CH_3_	OCH_3_	H	CH_3_	βCH_3_	βCH_3_	βCH_3_	VIII	[49]
**205**	5-*epi*-Nakijinone A	CH_3_	OCH_3_	H	CH_3_	αCH_3_	βCH_3_	βCH_3_	VIII	[49]
**206**	18-Deoxy-18-formamidodictyoceratin B	COOCH_3_	NHCHO	OH	βH	αCH_3_	αCH_3_	αCH_3_	IX	[50]
**207**	18-Deoxy-18-(2-hydroxyacetyl)aminodictyoceratin B	COOCH_3_	NHCOCH_2_OH	OH	βH	αCH_3_	αCH_3_	αCH_3_	IX	[50]
**208**	*N*-Methyl-ent-smenospongine	H	NHCH_3_	OH	βH	αCH_3_	αCH_3_	αCH_3_	I	[50]
**209**	*N*-Methyl-5-epi-smenospongine	H	NHCH_3_	OH	αH	αCH_3_	βCH_3_	βCH_3_	I	[50]
**210**	20-Demethoxy-20-methylaminodactyloquinone D	H	NHCH_3_	-	αH	βCH_3_	βCH_3_	βCH_3_	X	[50,54]
**211**	20-Demethoxy-20-methylamino-5-epidactylo-quinone D	H	NHCH_3_	-	αH	βCH_3_	βCH_3_	βCH_3_	IV	[50]
**212**	20-Demethoxy-20-methylaminodactyloquinone B	H	NHCH_3_	-	-	αCH_3_	βCH_3_	βCH_3_	XI	[50]
**213**	5-*epi*-Smenospongine	H	NH_2_	OH	αH	αCH_3_	βCH_3_	βCH_3_	IV	[50]
**214**	Smenospongiadine	H	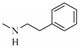	OH	αH	βCH_3_	βCH_3_	βCH_3_	IV	[50]
**215**	Dactylospongin A	H	OH	H	βH	αCH_3_	αCH_3_	αCH_3_	XII	[51]
**216**	Dactylospongin B	H	OH	H	αH	βCH_3_	βCH_3_	βCH_3_	XIII	[51]
**217**	Dactylospongin C	NHCHO	H	H	βH	αCH_3_	αCH_3_	αCH_3_	XIV	[51]
**218**	Dactylospongin D	NHCHO	H	H	αH	βCH_3_	βCH_3_	βCH_3_	XV	[51]
**219**	*ent*-Melemeleone B	NHCH_2_CH_2_SO_3_H	H	H	βH	αCH_3_	αCH_3_	αCH_3_	V	[51]
**220**	Melemeleone C	H	NHCH_2_CH_2_SO_3_H	H	βH	αCH_3_	αCH_3_	αCH_3_	V	[51]
**221**	Melemeleone D	NHCH_2_CH_2_SO_3_H	H	H	αH	βCH_3_	βCH_3_	βCH_3_	IV	[51]
**222**	Melemeleone E	H	NHCH_2_CH_2_SO_3_H	-	αH	βCH_3_	βCH_3_	βCH_3_	XVI	[51]
**223**	Dysidaminone N	H	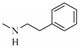	H	αH	βCH_3_	βCH_3_	βCH_3_	IV	[51]
**224**	Nakijiquinone V	H	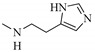	OH	αH	βCH_3_	βCH_3_	βCH_3_	IV	[52]
**225**	Cinerol A	H	OH	-	αH	βCH_3_	βCH_3_	βCH_3_	XVII	[53]
**226**	Cinerol B	H	OH	-	αH	βCH_3_	βCH_3_	βCH_3_	XVIII	[53]
**227**	Cinerol C	H	OH	H	αH	βCH_3_	βCH_3_	βCH_3_	VI	[53]
**228**	Cinerol D	H	OH	CH_3_	αH	βCH_3_	βCH_3_	βCH_3_	VI	[53]
**229**	Cinerol E	H	OH	H	CH_3_	βCH_3_	βCH_3_	βCH_3_	II	[53]
**230**	Cinerol F	H	OH	H	αH	βCH_3_	βCH_3_	βCH_3_	XIX	[53]
**231**	Cinerol G	H	OH	CH_3_	αH	βCH_3_	βCH_3_	βCH_3_	XIX	[53]
**232**	Cinerol H	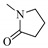	H	H	αH	βCH_3_	βCH_3_	βCH_3_	XIV	[53]
**233**	Cinerol I	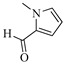	H	H	αH	βCH_3_	βCH_3_	βCH_3_	XIV	[53]
**234**	Cinerol J	NHCHO	H	H	αH	βCH_3_	βCH_3_	βCH_3_	XIV	[53]
**235**	Cinerol K	NHCOCH_2_CH(CH_3_)_2_	H	H	αH	βCH_3_	βCH_3_	βCH_3_	XIV	[53]
**236**	20-Demethoxy-20-isopentylaminodactyloquinone D	H	NH(CH_2_)_2_CH(CH_3_)_2_	-	αH	βCH_3_	βCH_3_	βCH_3_	X	[54]
**237**	20-Demethoxy-20-isobutylaminodactyloquinone D	H	NHCH_2_CH(CH_3_)_2_	-	αH	βCH_3_	βCH_3_	βCH_3_	X	[54]
**238**	Smenospongiarine	H	NH(CH_2_)_2_CH(CH_3_)_2_	OH	βH	αCH_3_	αCH_3_	αCH_3_	I	[54]
**239**	Smenospongorine	H	NHCH_2_CH(CH_3_)_2_	OH	βH	αCH_3_	αCH_3_	αCH_3_	I	[54]
**240**	Smenospongimine	H	NHCH_3_	OH	βH	αCH_3_	αCH_3_	αCH_3_	I	[54]
**241**	(+)-19-Methylaminoavarone	NHCH_3_	H	H	αH	βCH_3_	βCH_3_	βCH_3_	V	[55]
**242**	(−)-20-Phenethylaminoavarone	H	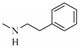	H	αH	βCH_3_	βCH_3_	βCH_3_	V	[55]
**243**	(−)-20-Methylaminoavarone	H	NHCH_3_	H	αH	βCH_3_	βCH_3_	βCH_3_	V	[55]
**244**	Dysidinoid B	H	H	-	αH	βCH_3_	βCH_3_	βCH_3_	XX	[56]
**245**	Dysicigyhone A	H	OH	CH_3_	αH	βCH_3_	βCH_3_	βCH_3_	XXI	[56]
**246**	5-*epi*-Nakijiquinone L	H	NHCH_2_CH(CH_3_)CH_2_CH_3_	OH	αH	αCH_3_	βCH_3_	βCH_3_	IV	[57]
**247**	5-*epi*-Smenospongiarine	H	NH(CH_2_)_2_CH(CH_3_)_2_	OH	αH	αCH_3_	βCH_3_	βCH_3_	IV	[57]

**Table 3:** The species containing nitrogenous sesquiterpenoids.

Classification	Family	Species	Type	Reference
Plant	Celastraceae	*Maytenus mekongensis; M. spinosa; M. oblongata*	Dihydroagarofuran	[17,25,30]
		*Tripterygium wilfordii; T. regelii; T. hypoglaucum*		[18,20,21,23,24,26,28,29,31,32,33,36]
		*Celastrus orbiculatus; C. angulatus; C. paniculatus*		[19,34,37]
		*Euonymus alatus*		[22]
		*Monimopetalum chinense*		[35]
	Saxifragaceae	*Parnassia wightiana*		[27]
	Zingiberaceae	*Curcuma phaeocaulis*	Eudesmane; Elemene	[63]
	Asteraceae	*Inula helenium* L.	Eudesmane	[68]
		*Onopordum alexandrinum*	Germacrane; Elemene	[79]
		*Vladimiria souliei*	Guaiane	[93]
	Burseraceae	*Resina commiphora*	Cadinane	[72]
	Phyllanthaceae	*Phyllanthus acidus* (L.) skeels	Bisabolane	[75]
	Magnoliaceae	*Magnolia kobus*	Germacrane	[76]
	Lamiaceae	*Salvia scapiformis*	Germacrane	[78]
	Myoporaceae	*Myoporum bontioides*	Farnesane	[83]
	Valerianaceae	*Valeriana officinalis* var. *latifolia*	Valerane	[88]
		*Nardostachys chinensis*	Nornardosinane-aristolane	[92]
Sponge	Dysiseidae	*Dysidea* sp.; *D. avara; D. fragilis; D. cinerea; D. septosa*	friedo-drimane	[43,45,48,53,55,56]
	Thorectidae	*Dactylospongia* sp.; *D. elegans*; *D. metachromia*		[44,47,51,52,54]
		*Smenospongia aurea*, *S. cerebriformis*, *and Verongula rigida*		[49]
		*Verongula* cf. *rigida* Esper		[57]
	Spongiidae	*Hippospongia* sp.		[46]
		*Spongiapertusa* Esper		[50]
	Halichodriae	*Halichondria* sp.; *H. okadai*	Eudesmane; Cadinane; Spiroaxane; Aromadendrane; Bisabolane; Pupukeanane; Salvialane; Aristolane; Iresane	[60,61,62,65,69,74,77]
		*Axinyssa* sp.; *A. variabilis*	Eudesmane; Cadinane; Bisabolene	[66,70]
	Thorectidae	*Fasciospongia* sp.	Farnesane	[82]
Soft coral	Xeniidae	*Cespitularia taeniata*	Eudesmane	[64]
	Clavulariidae	*Clavularia koellikeri*	Nardosinane	[94]
Phyllidid nudibranchs	Phyllidiidae	*Phyllidiella* sp.; *P. pustulosa*; *P. ocellata*	Eudesmane; Cadinane; Bisabolane; Farnesane, spiroaxane; aromadendrane; pupukeanane; Axane	[67,71]
Marine slug	Dotidae	*Doto pinnatifida*	Farnesane	[81]
Fungus	Trichocomaceae	*Aspergillus ochraceus; A. aculeatus*	Drimane; Daucane	[38,39,85]
		*Talaromyces minioluteus*	Drimane	[40]
		*Emericella* sp.	Farnesane	[80]
	Eurotiaceae	*Penicillium* sp. ZZ1283.	Drimane	[41]
	Parmulariaceae	*Paraconiothynium brasiliense*; *P. sporulosum*	Bisabolane; Bergamotane	[73,87]
	Phanerochaetaceae	*Ceriporia lacerate*	Tremulane	[84]
	Diaporthaceae	*Diaporthe* sp.	Brasilane	[86]
	Moniliaceae	*Trichoderma asperellum*	Cyclonerane	[89]
	Pezizaceae	*Cochliobolus lunatus*	Eremophilane	[91]
Bacteria	Pseudomonadaceae	*Saccharomonospora* sp. CNQ-490	Drimane	[42]
Actinomyces	Streptomycetaceae	*Streptomyces* sp.	Drimane; Zizaane	[58,59,90]

## Abbreviations

OAc	
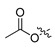
**OBz**	
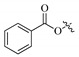
OFu	
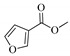
**ONic**	
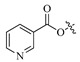
**O*t*Cin**	
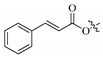
**O*c*Cin**	
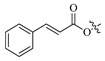
**OTig**	
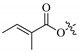
**OMeBut**	
